# Identification of subgroup-specific miRNA patterns by epigenetic profiling of sporadic and Lynch syndrome-associated colorectal and endometrial carcinoma

**DOI:** 10.1186/s13148-015-0059-3

**Published:** 2015-03-10

**Authors:** Sippy Kaur, Johanna E Lotsari, Sam Al-Sohaily, Janindra Warusavitarne, Maija RJ Kohonen-Corish, Päivi Peltomäki

**Affiliations:** Department of Medical Genetics, Biomedicum Helsinki, University of Helsinki, Haartmaninkatu 8, P.O. Box 63, Helsinki, FIN-00014 Finland; The Kinghorn Cancer Centre, Garvan Institute of Medical Research, 370 Victoria Street, Darlinghurst, Sydney, NSW 2010 Australia; St Vincent’s Clinical School, UNSW Medicine, Darlinghurst, NSW 2052 Australia; School of Medicine, University of Western Sydney, Campelltown, Sydney, NSW 2560 Australia

**Keywords:** miRNA, Methylation, Microsatellite instability, Lynch syndrome, Colorectal cancer, Endometrial cancer

## Abstract

**Background:**

Altered expression of microRNAs (miRNAs) commonly accompanies colorectal (CRC) and endometrial carcinoma (EC) development, but the underlying mechanisms and clinicopathological correlations remain to be clarified. We focused on epigenetic mechanisms and aimed to explore if DNA methylation patterns in tumors depend on DNA mismatch repair (MMR) status, sporadic *vs*. Lynch-associated disease, and geographic origin (Finland *vs*. Australia). Treatment of cancer cell lines with demethylating agents revealed 109 significantly upregulated miRNAs. Seven met our stringent criteria for possible methylation-sensitive miRNAs and were used to screen patient specimens (205 CRCs and 36 ECs) by methylation-specific multiplex ligation-dependent probe amplification.

**Results:**

Three miRNAs (129-2, 345, and 132) with low methylation levels in normal tissue and frequent hypermethylation in tumors were of particular interest. Hypermethylation of miR-345 and miR-132 associated with MMR deficiency in CRC regardless of geographic origin, and hypermethylation of miR-132 distinguished sporadic MMR-deficient CRC from Lynch-CRC. Finally, hypermethylation of miRNAs stratified 49 endometrial hyperplasias into low-methylator (simple hyperplasia) and high-methylator groups (complex hyperplasia with or without atypia) and suggested that miR-129-2 methylation in particular could serve as a marker of progression in early endometrial tumorigenesis.

**Conclusions:**

Our study identifies miR-345 and miR-132 as novel differentially methylated miRNAs in CRC, thereby facilitating sub-classification of CRC and links miR-129-2 methylation to early endometrial tumorigenesis.

**Electronic supplementary material:**

The online version of this article (doi:10.1186/s13148-015-0059-3) contains supplementary material, which is available to authorized users.

## Background

MicroRNAs (miRNAs) are small non-coding RNAs that control gene expression by binding to complementary sequences in the 3′UTR of target mRNAs, thereby inducing mRNA degradation or translational repression [1]. More than 1,400 human miRNAs are known [2], and these may regulate about one third of all human genes [3]. Approximately half of mammalian miRNAs are intergenic and transcribed independently, whereas another half are intragenic (located within introns of host genes) and can be, but not necessarily are, co-transcribed with their host genes [4].

Downregulation of tumor suppressive miRNAs (target proto-oncogenes) and upregulation of oncogenic miRNAs (target tumor suppressor genes) is a feature of cancer [5]. Over half of miRNA promoters contain a CpG island as a possible target for aberrant methylation ^6^[6] which can lead to miRNA dysregulation. The CpG island is typically located in the proximal upstream region (<2 kb of pre-miRNAs) for intergenic miRNAs and independently regulated intronic miRNAs, but can reside far upstream (>20 kb of pre-miRNAs) for intronic miRNAs utilizing the host transcription start sites [6-8]. Epigenetically silenced miRNAs can be reactivated in cancer cell lines by treatment with the DNA methyltransferase inhibitor 5-aza-2′-deoxycytidine (5-aza-CdR), often combined with a histone deacetylase inhibitor (such as trichostatin A, TSA), and analogous drugs can be used for epigenetic cancer therapy in patients [9].

Colorectal cancer (CRC) can develop via two main routes, the microsatellite instability (MSI) pathway (approximately 15% of CRCs), or the chromosomal instability (CIN) pathway (approximately 85% of CRCs) [10]. The most frequent cause of MSI is somatic methylation of the DNA mismatch repair (MMR) gene *MLH1* and involves the CpG island methylator phenotype (CIMP). Lynch syndrome (LS), which is associated with germline mutations in one of four MMR genes, *MLH1*, *MSH2*, *MSH6*, and *PMS2*, underlies one fifth of MSI cancers [10]. In LS, tumor suppressor methylator phenotypes vary between cancers arising in different organs, and the patterns are partly different compared to the corresponding sporadic cancers [11,12].

This study took advantage of principles described above to search for novel epigenetically silenced tumor-suppressive miRNAs that might play a role in colorectal and endometrial tumorigenesis and discriminate tumors according to their molecular characteristics (MMR status and sporadic *vs*. Lynch-associated disease), geographic origin (Finland *vs*. Australia), and developmental stage (benign *vs*. malignant histology). Results of seven miRNAs, including three of particular interest (miR-129-2, miR-345, and miR-132) will be reported.

## Results

### Study design

**Figure 1:**
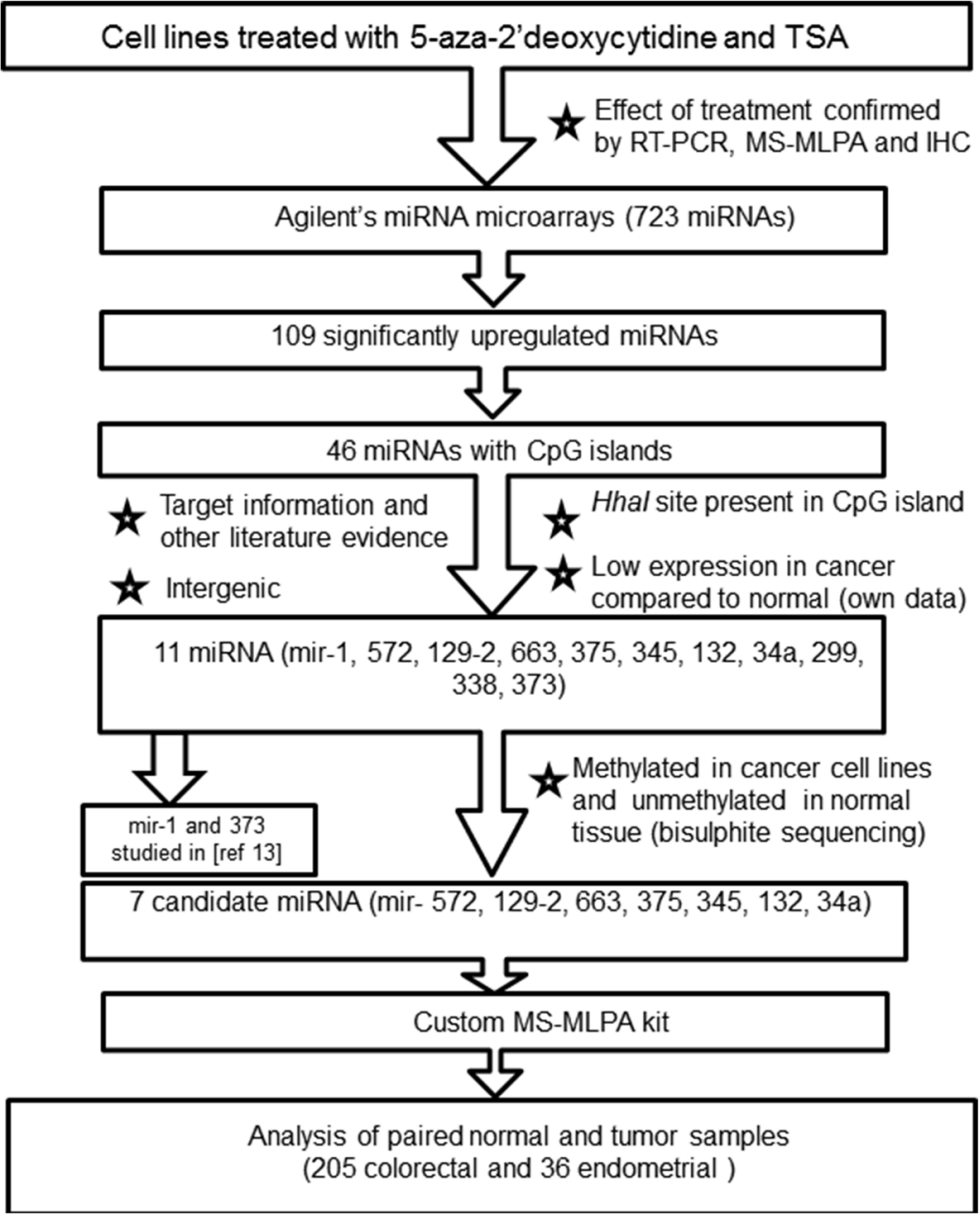
**Flow chart of this investigation.**

To identify methylation-sensitive miRNAs associated with colorectal and endometrial tumorigenesis, cancer cell lines and clinical specimens were utilized according to a scheme depicted in Figure 1. The cell lines (Additional file 1: Table S1) were selected to represent the main molecular subtypes of the patient series (Table 1) with the MMR-proficient SW480 and T84 corresponding to sporadic microsatellite stable (MSS) colorectal cancers, RKO and AN3CA (MMR-deficient due to presumably biallelic *MLH1* promoter methylation) equivalent to sporadic MSI cancers and HCT15, HCT116, and HEC59 (MMR-deficient as a result of MMR gene mutations) analogous to LS-CRC and LS-EC. While cell lines tend to have stronger methylator phenotypes than primary tumors, the basic patterns of CIMP are often broadly comparable in cell lines and primary tumors, including tissue-specific involvement of marker loci and relationship to genomic instability [11,13-15]. Criteria to select miRNAs from cell line experiments for the subsequent screen of clinical samples (Figure 1) were based on bioinformatic and literature analyses on the one hand (associated with CpG island; predicted to target genes relevant in colorectal and endometrial tumorigenesis; expressed in the intestine and endometrium; intergenic; *HhaI* site present in CpG island) and our own experimental data on the other hand (low expression in cancer cell lines compared to respective normal tissues; methylated in the cell lines before treatment and showing significant upregulation along with reduced methylation of various degrees after treatment). Altogether, 109 miRNAs were upregulated at least twofold in one or more cell lines (Additional file 2: Figure S1). Among those, 46 were associated with CpG islands. Among these, seven miRNAs with a high *a priori* probability of representing novel methylation-sensitive, intergenic/independently regulated tumor-suppressive miRNAs according to the abovementioned criteria were chosen for the analysis of patient specimens.

**Table 1:** **Basic molecular and clinicopathological characteristics of the study series**

	**Finnish CRC**			**Australian CRC**		**Finnish EC**
	**Sporadic MSS**	**Sporadic MSI**	**Lynch**	**Sporadic MSS**	**Sporadic MSI**	**Lynch**
Total number of tumors	47	40	28	52	38	36
Gender						
Female	27	27	12	25	20	36
Male	20	13	16	27	18	-
Mean age of diagnosis (years)	69.9	72.8	43.6	67.6	67.1	50.2
Germline mutation present in MMR genes (total)	N/A	N/A	28	N/A	N/A	36
*MLH1*			28			30
*MSH2*			0			3
*MSH6*			0			3
MMR status^a^						
Deficient	0	40	28	0	38	36
Proficient	47	0	0	52	0	0
Tumor grade						
1	13/21	5/14	5/16	1/52	0/36	14/29
2	5/21	6/14	6/16	46/52	24/36	9/29
3	3/21	3/14	5/16	5/52	12/36	6/29
Tumor stage (Dukes/WHO/FIGO)^b^						
A/I/I	7/46	2/37	5/19	8/52	3/38	12/22
B/II/II	16/46	24/37	10/19	24/52	20/38	7/22
C/III/III	16/46	8/37	4/19	17/52	13/38	1/22
D/IV/IV	7/46	3/37	0/19	3/52	2/38	3/22
Location of CRC^c^						
Proximal	20/44	34/39	22/27	20/52	27/38	-
Distal	24/44	5/39	5/27	32/52	11/38	-

N/A, not applicable. ^a^Based on microsatellite instability and immunohistochemical staining; ^b^according to the Dukes (A-D), World Health Organization (WHO) (I-IV), and International Federation of Gynecology and Obstetrics (FIGO) (I-IV) staging for CRC and EC, respectively. The denominator indicates the number of tumors for which data were available. ^c^Prior to the splenic flexure for ‘proximal’ and distal to the splenic flexure for ‘distal’ (the denominator indicates the number of tumors with data available).

### Aberrant methylation of miRNA-associated CpG islands in CRC and EC

**Figure 2:**
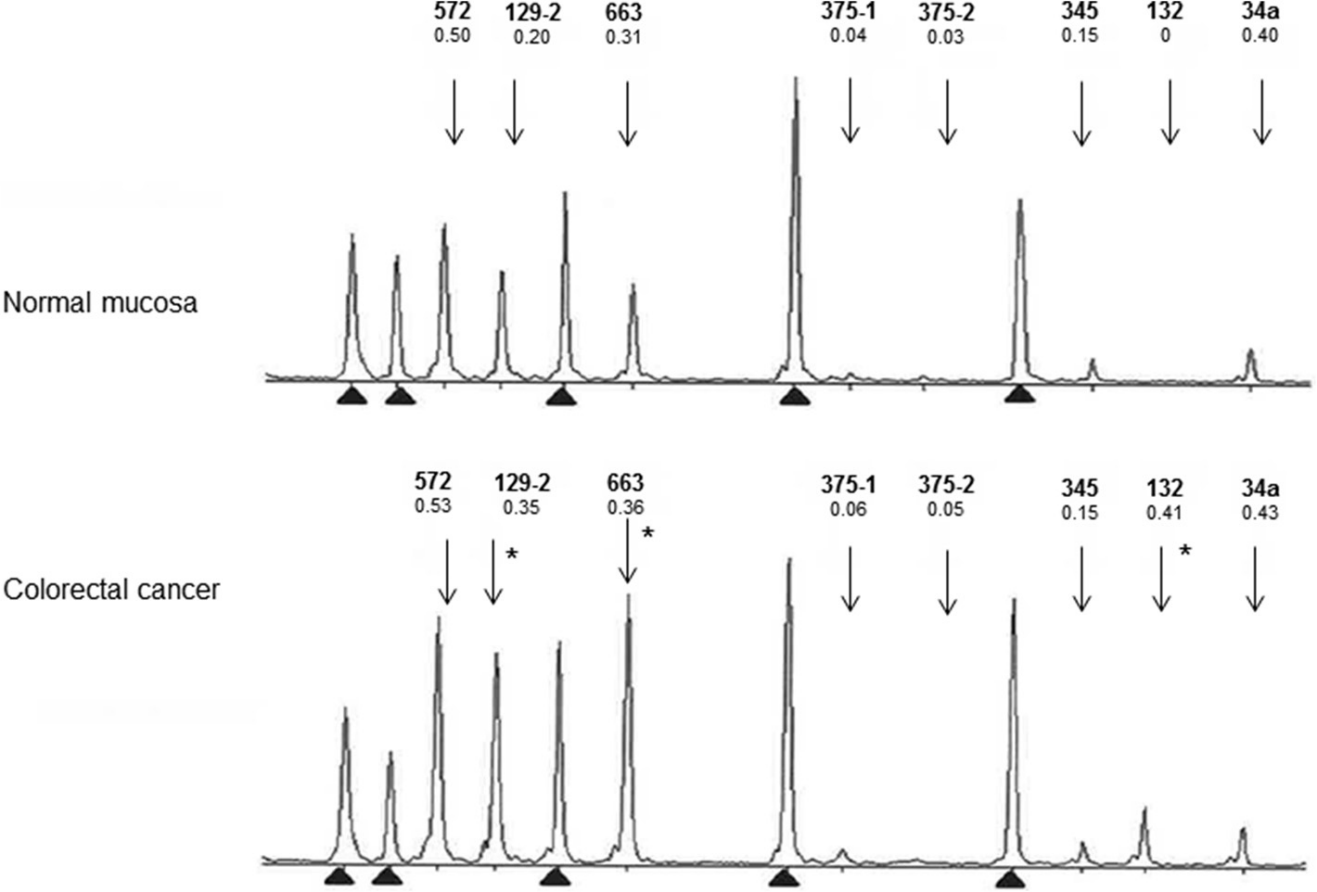
**Electropherograms for paired normal and tumor DNA to illustrate a typical output from the custom-made MS-MLPA test.** A single case from the Finnish MSI-CRC group is shown. Dm values obtained for each miRNA locus are given below the name of the miRNA. Arrows denote the peak positions of the miRNAs which reflect the sizes of the amplified fragments (Additional file 5: Table S3). Based on cutoff values for hypermethylation derived from this series (Additional file 8: Table S5), the tumor shows hypermethylation at miRNA loci 129-2, 663, and 132 (asterisks). Reference peaks are indicated by arrowheads.

**Figure 3:**
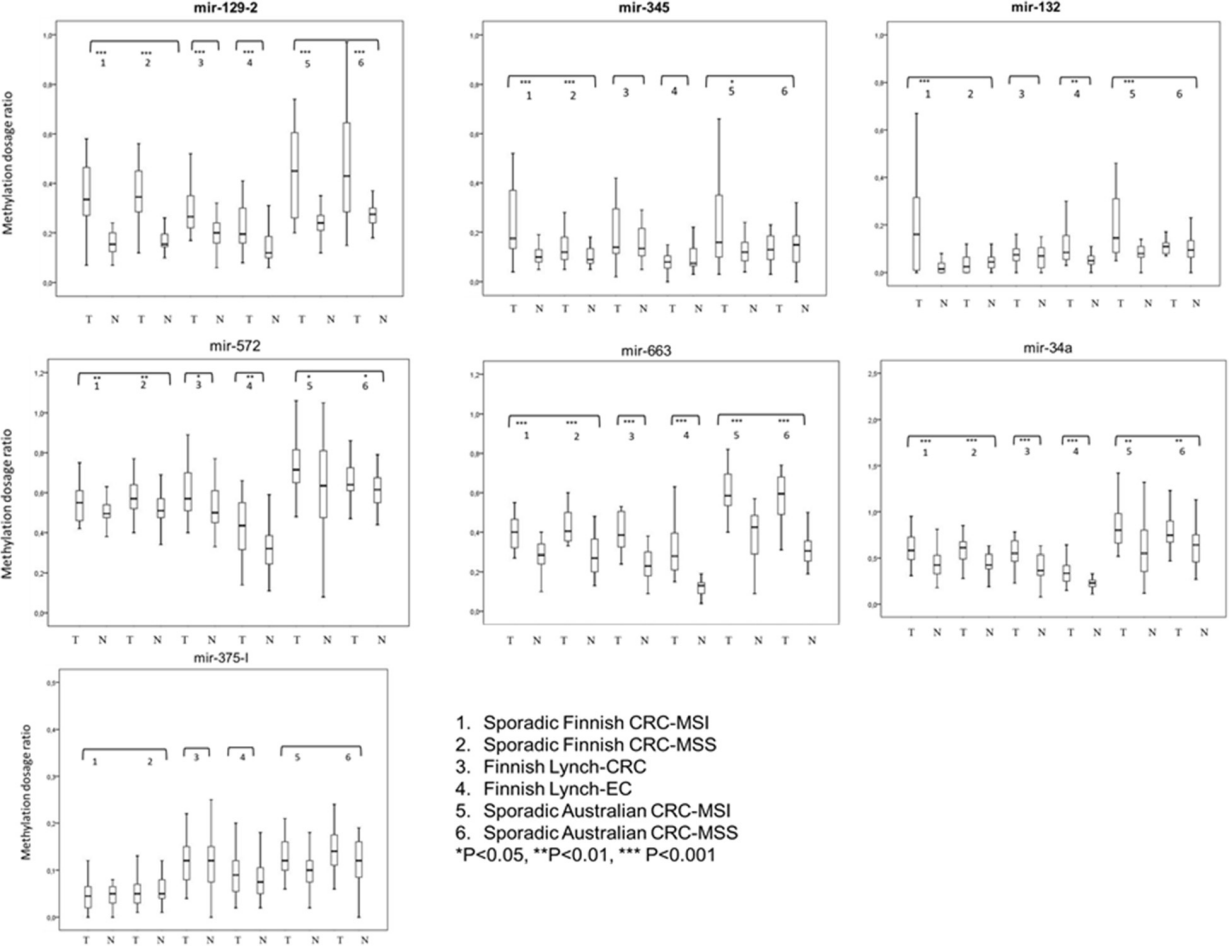
**Box-and-whisker plots of miRNA-specific distributions of methylation dosage ratios.** The Dm values in paired tumor and normal DNAs from each patient group are shown (please see the key for details). The horizontal line inside the box denotes the median, the upper and lower edges are the 75th and 25th percentiles, and the bars indicate the highest and lowest Dm values. *P* values are based on *t*-test for paired samples or Wilcoxon signed rank test. The miRNAs 129-2, 345, and 132 are shown in bold because their methylation pattern was considered the most promising for further studies.

The seven miRNAs of interest were investigated for CpG island methylation (Additional file 3: Figure S2, Additional file 4: Table S2, Additional file 5: Table S3) in clinical specimens (205 CRC and 36 EC, Table 1) to explore if methylation in tumor DNA differs from that in paired normal DNA and if patterns specific to individual patient groups might be detectable. We opted for a methylation-specific multiplex ligation-dependent probe amplification (MS-MLPA)-based approach, which allows for multiplex, quantitative analysis of methylation in archival formalin-fixed, paraffin-embedded (FFPE) specimens without the need of bisulfite conversion [16]. A representative electropherogram from MS-MLPA analysis is shown in Figure 2. MS-MLPA data from normal and tumor DNAs from all 241 cases, divided into six patient groups, are shown in Figure 3 (box plots of distributions of individual Dm values) and Additional file 6: Table S4 (averages and standard deviations of Dm values). Three different methylation patterns emerged. First, miR-129-2, 345, and 132 showed low levels of methylation in normal DNA (with average Dm values around 0.20 or below) and increased methylation in tumor DNA; the difference was statistically significant in most patient groups. Second, miR-572, 663, and 34a had considerable methylation in normal DNA already (with average Dm values clearly exceeding 0.20), and methylation increased in tumor DNA. Third, miR-375 showed little methylation (for both probe I and probe II) and no difference between normal and tumor DNA. The pattern displayed by the first set of miRNAs was considered to have the highest biomarker potential and became our main focus in subsequent analyses.

Since the baseline levels of methylation may vary depending on the locus and tissue type (Additional file 7: Figure S3), thresholds for hypermethylation were determined for each miRNA based on Dm values in the respective normal tissues (Methods, Additional file 8: Table S5). Percentages of tumors with hypermethylation (Additional file 7: Figure S3) were then used to compare the different patient groups (Table 2). Among the three miRNAs of our primary interest, miR-345 and 132 showed hypermethylation in a significantly higher percentage of MSI tumors (60% and 58%, respectively) than MSS tumors (28% and 2%, respectively) from the Finnish sporadic CRC series. Examination of Australian CRCs provided independent additional proof by demonstrating that hypermethylation of miR-345 and miR-132 significantly associated with MSI. Furthermore, hypermethylation percentages of miR-132 distinguished Finnish sporadic MSI-CRCs from Lynch-CRC (58% *vs*. 7%, *P* < 0.001). Hypermethylation of miR-129 and 345, respectively, was significantly (*P* < 0.05) more common in Lynch-CRC (82% and 43%) than that in Lynch-EC (44% and 8%), emphasizing tissue specificity of methylation patterns. Finally, hypermethylation frequencies of several miRNAs differed between Finnish and Australian CRCs, suggesting that methylation patterns depend on geographic origin.

**Table 2:** **Comparison of tumors from different patient groups based on frequencies of hypermethylation at miRNA loci**

	**Proportion of tumors with hypermethylation** ^**a**^						
**Tumor category**	**572**	**129-2**	**663**	**375-I**	**345**	**132**	**34a**
MSI *vs*. MSS							
Sporadic Finnish MSI-CRC (*n* = 40) *vs*. sporadic Finnish MSS-CRC (*n* = 47)	13/40 *vs*. 13/47	37/40 *vs*. 34/47	28/40 *vs*. 28/47	0/40 *vs*. 1/47	24/40 *vs*. 13/47*	23/40 *vs*. 1/47***	23/40 *vs*. 20/47
Sporadic Australian MSI-CRC (*n* = 38) *vs*. sporadic Australian MSS-CRC (*n* = 52)	9/38 *vs*. 9/52	28/38 *vs*. 42/52	30/38 *vs*. 44/52	10/38 *vs*. 14/52	13/38 *vs*. 5/52*	15/38 *vs*. 6/52*	10/38 *vs*. 15/52
Sporadic *vs*. hereditary							
Sporadic Finnish MSI-CRC (*n* = 40) *vs*. Finnish Lynch-CRC (*n* = 28)	13/40 *vs*. 12/28	37/40 *vs*. 23/28	28/40 *vs*. 18/28	0/40 *vs*. 8/28**	24/40 *vs*. 12/28	23/40 *vs*. 2/28***	23/40 *vs*. 13/28
Colorectal *vs*. endometrial							
Finnish Lynch-CRC (*n* = 28) *vs*. Finnish Lynch-EC (*n* = 36)	12/28 *vs*. 16/36	23/28 *vs*. 16/36*	18/28 *vs*. 22/36	8/28 *vs*. 5/36	12/28 *vs*. 3/36*	2/28 *vs*. 11/36	13/28 *vs*. 20/36
Finnish *vs*. Australian							
Sporadic Finnish MSI-CRC (*n* = 40) *vs*. sporadic Australian MSI-CRC (*n* = 52)	13/40 *vs*. 9/38	37/40 *vs*. 28/38	28/40 *vs*. 30/38	0/40 *vs*. 10/38**	24/40 *vs*. 13/38	23/40 *vs*. 15/38	23/40 *vs*. 10/38*
Sporadic Finnish MSS-CRC (*n* = 47) *vs*. sporadic Australian MSS-CRC (*n* = 52)	13/47 *vs*. 9/52	34/47 *vs*. 42/52	28/47 *vs*. 44/52*	1/47 *vs*. 14/52**	13/47 *vs*. 5/52	1/47 *vs*. 6/52	20/47 *vs*. 15/52

^a^Using cutoffs determined by methylation in the respective normal tissues (Additional file 8: Table S5). *P* values determined by Fisher’s exact test and adjusted for multiple testing are shown after each comparison, (* *P* < 0.05; ** *P* < 0.01; *** *P* < 0.001). All tumors were informative for all markers.

To evaluate if methylation of the miRNAs was part of a more generalized methylator phenotype, the Dm values of individual miRNAs were assessed against the proportion of conventional tumor suppressor gene (TSGs) [11,16] or CIMP markers methylated [17]. Methylation of miR-132 was significantly (*P* < 0.001 by Spearman analysis) correlated with TSG methylation (Finland) or CIMP (Australia) in sporadic MSI CRCs. Moreover, to prove that the observed correlations did not depend on any particular definition of a methylator phenotype, the Finnish tumors were additionally examined with the same CIMP markers used to classify Australian tumors [17], and the conclusion remained unaltered (*P* = 0.006 for the correlation between miR-132 methylation and CIMP). No significant correlation was present in sporadic MSS CRCs or Lynch-CRCs. The remaining miRNAs mostly did not show significant correlations with a methylator phenotype.

For analyses of clinical correlations, we evaluated possible associations of the Dm values for miR-129-2, 345, and 132 with gender, age, grade, stage, and sidedness among sporadic CRCs stratified by the MMR status. In the combined series of CRCs from Finland and Australia, the MSI CRCs revealed a significant association between miR-132 methylation and female gender (average Dm was 0.28 in females, *n* = 47, *vs*. 0.14 in males, *n* = 31; *P* = 0.003), increased age (across four age groups; *P* = 0.047), and proximal location in the bowel (average Dm was 0.21 in proximal CRC, *n* = 61, *vs*. 0.11 in distal CRC, *n* = 16; *P* = 0.000) when analyzed by the Kruskal-Wallis test. MSS CRCs did not show significant differences in these respects. For all three miRNAs, the average Dm value for poorly differentiated (grade 3) CRCs exceeded that of well-differentiated (grade 1) tumors regardless of the MMR status, but the grade associations did not reach statistical significance. Methylation of the miRNAs did not correlate with stage.

### Aberrant methylation of miRNA-associated CpG islands in endometrial hyperplasia

**Figure 4:**
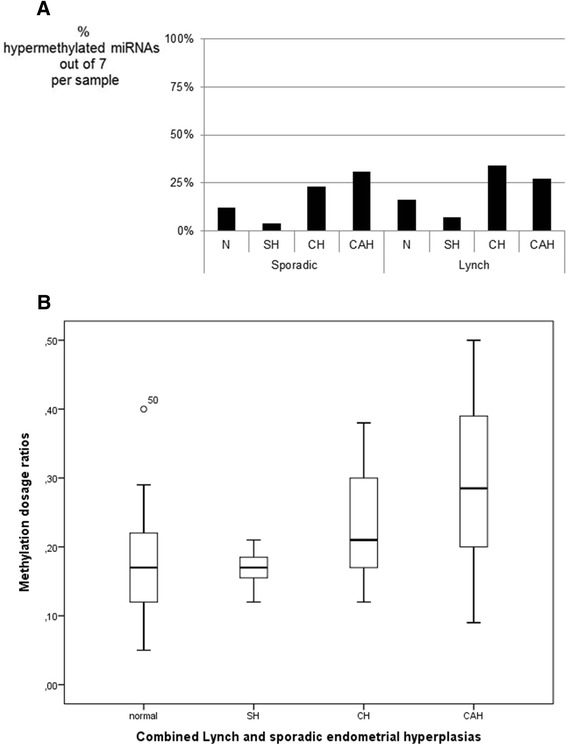
**Average percentages of hypermethylated miRNAs and box-and-whisker plots. (A)** Average percentages of hypermethylated miRNAs out of 7 per endometrial sample. The set of miRNAs include miR-572, 129-2, 663, 375-I, 345, 132, and 34a. Specimens of normal endometrium (N) and endometrial hyperplasias of increasing severity (SH, CH, and CAH) are displayed. Sporadic and Lynch syndrome-associated cases are shown separately (see the ‘Methods’ section for sample sizes). **(B)** Box-and-whisker plots for the distribution of Dm values for 129-2 in normal endometrium and endometrial hyperplasias. Sporadic and Lynch syndrome-associated cases were combined since they revealed similar distributions when analyzed individually.

To address the developmental stage at which epigenetic alterations may arise, 29 normal endometrial tissues and 49 endometrial hyperplasias were screened for aberrant methylation of the seven miRNAs of interest. Thresholds for hypermethylation were determined on the basis of Dm values in normal endometrial tissues (for sporadic and Lynch cases separately, see Additional file 8: Table S5), and the mean number of miRNAs with hypermethylation (out of 7) per sample was calculated for the different types of endometrial lesions (Figure 4A). In both sporadic and Lynch series, normal endometrium and simple hyperplasia (SH) clustered together to form a low-methylator group, whereas complex hyperplasia without atypia (CH) and complex hyperplasia with atypia (CAH) jointly constituted a high-methylator group. When sporadic and Lynch cases were combined, the low-methylator group (normal endometrium + SH) had an average of 0.80 miRNAs with hypermethylation out of 7 (11%), compared to 2.0 (28%) in the high-methylator group (*P* = 0.00017 by *t*-test for independent samples).

Among the individual miRNAs, miR-129-2 was the most discriminative between the different types of endometrial lesions (Figure 4B) and displayed a progressively increasing methylation from normal endometrium and SH to CH and CAH (the mean Dm was 0.17 for normal endometrium and SH *vs*. 0.28 for CH and CAH in the combined sporadic and Lynch series, *P* < 0.0001 by *t*-test for independent samples). Nine ECs were diagnosed in the Lynch syndrome patients with hyperplasias, and the average Dm was 0.25 in these ECs (data not shown).

## Discussion

The available knowledge of cancer associations of miRNAs is mainly derived from cell lines and unselected sporadic forms of cancer. This study combined experiments on cell lines and clinical specimens of sporadic and hereditary CRCs and ECs to identify novel epigenetically regulated miRNAs with translational relevance. Three miRNAs (miR-129-2, miR-345, and miR-132) emerged as promising targets for subgroup-specific methylation in CRC and EC in our expressional screen and subsequent methylation profiling of patient specimens (Figure 1).

To our knowledge, our study is the first to report miR-132 as a methylation target in CRC and EC. Recent studies described hypermethylation of miR-132 in cancers of the pancreas (hypermethylation frequency not given [18]) and prostate in 42% [19]. Downregulation of miR-132 has been observed in CRC tumors with distant metastases [20]. In our investigation, hypermethylation of the miR-132-associated CpG island (in up to 58% of CRCs) was associated with sporadic MSI CRC as opposed to MSS CRC and distinguished sporadic MSI CRC from Lynch-CRC (Table 2). Among sporadic MSI CRCs, miR-132 methylation was a particular property of tumors located in the proximal colon (the average level of methylation in distal MSI CRCs was similar to that in MSS CRCs irrespective of location). Recent reports suggest that the CIMP status, and not MSI, may primarily determine the response to adjuvant chemotherapy [21]. Given that hypermethylation of miR-132 was found to be part of a generalized TSG methylator/CIMP phenotype, our findings imply that treatment of MSI CRCs may need to be tailored according to the proximal *vs*. distal location of the tumor.

MiR-345 was another miRNA showing differential methylation between the various clinical groups examined (Table 2). While our study was in progress, Tang et al. [22] reported hypermethylation of miR-345 in 87% (27/31) of primary colorectal cancers by methylation-specific PCR. When quantified by bisulfite genomic sequencing, the levels of methylation were relatively low (16% for CRCs on the average), yet significantly higher than in the paired non-cancerous mucosa (8.7% [22]). In our MS-MLPA-based approach, a methylation dosage ratio of 0.18 (corresponding to a methylation level of 18%) was used as the cut-off value for hypermethylation of miR-345 in Finnish CRCs and a significant association with sporadic MSI (as opposed to MSS) CRC emerged, with a hypermethylation frequency of 60% (Table 2) and average Dm 0.26 (Additional file 6: Table S4). Evaluation of Australian CRCs confirmed the observed association of miR-345 with MSI (Table 2).

Our findings regarding miR-129-2 were important in two respects. First, miR-129-2 displayed the highest frequencies of hypermethylation among all seven miRNAs analyzed (93% for sporadic Finnish CRC, Table 2). Second, in a series of endometrial hyperplasias, hypermethylation of miR-129-2 progressively increased along with the increasing severity of the lesion (Figure 4B). Published studies indicate that hypermethylation of the miR-129-2-associated CpG island is common in human cancers, occurring in 91% of CRC [23], 69% of gastric cancers [24], 68% of EC [25], and 58% of hepatocellular carcinomas [26]. Moreover, correlation of hypermethylated miR-129-2 with MSI, *MLH1* methylation, and poor survival in EC has been reported [25]. Earlier studies have not examined the occurrence of miR-129-2 hypermethylation in steps preceding malignant transformation. We showed that miR-129-2 methylation stratified endometrial specimens into two groups, one with low methylation (normal endometrium and SH) and another one with a significantly higher methylation (CH and CAH). It remains controversial which lesions should be considered as precursors to EC, and the miRNA data, together with our previous findings of conventional TSGs [27], support the importance of complex hyperplasia, with or without atypia, as a precursor lesion of EC.

Existing literature is scarce regarding the role of ethnic or geographic origin as a modifier of epigenetic patterns in cancer. While the causes for the significant differences in hypermethylation frequencies between sporadic CRCs from Finland and Australia (Table 2) are unknown, different environments [28] or population-specific features of the genetic constitution [29] could play a role. We recently described distinct epigenetic signatures of conventional TSGs for CRCs from Finland and Egypt, suggesting the possible effect of environmental exposures on colorectal carcinogenesis [30]. The selective targeting hypothesis for CIMP [31] postulates that certain regions of the genome may have intrinsic features that attract DNA methyltransferases: for example, a common polymorphism in the *MLH1* promoter region (−93G > A) was shown to increase the risk of MSI colon cancer with CIMP whereas MSS-CRCs did not show this association [32]. It is possible that the promoter regions of the miRNAs we studied harbor genetic variation between populations. However, the genetic homogeneity of the Finnish series (exclusively of Finnish origin) but considerable heterogeneity of the Australian series (of diverse ethnic origins) may make a genetic explanation less likely compared to the impact of different environments.

Multiple techniques are available for quantitative and qualitative analysis of methylation, each with advantages and disadvantages [33,34]. MethyLight, pyrosequencing, COBRA, and MS-MLPA represent methods suitable for quantitative methylation analysis for biomarker purposes. No need of bisulfite conversion distinguishes MS-MLPA from the other methods mentioned above. This is a clear advantage with respect to FFPE samples in particular which may result in non-reproducible bisulfite conversions and hence varying methylation levels [35]. The simultaneous analysis of multiple genes in a single assay saves template DNA and is another advantage of MS-MLPA over the remaining techniques. A drawback is that MS-MLPA only assesses one or two CpG sites (recognized by *HhaI*) for any given gene, whereas pyrosequencing allows quantitative analysis of methylation at multiple CpG sites [36-39]. This disadvantage of MS-MLPA can be alleviated by choosing CpG sites with methylation status reflecting that of the surrounding CpGs (this study, [40]); by doing so, methylation levels by MS-MLPA and pyrosequencing have turned out to be concordant [40]. In comparison with bisulfite sequencing, MS-MLPA and pyrosequencing share the advantage of not requiring any cloning step for accurate quantification of methylation. A good agreement between Dm values from MS-MLPA and proportions of methylated DNA by cloning is evident from previous studies [16,41] and this investigation (Additional file 9: Figure S4). Finally, MS-MLPA and pyrosequencing are both sensitive, being able to detect low percentages of methylated DNA (10% to 15% or even below; [36,42]) and specific when evaluated against results obtained by independent methods (this study, [36,39]).

CpG island methylation and expression of mature miRNAs, including miR-129-2 [26], miR-345 [22], and miR-132 [18] are inversely correlated suggesting that methylation is functionally significant. Inverse trends for these miRNAs were also evident in the colorectal and endometrial cancer cell lines and respective normal tissues we investigated, although the trends failed to reach statistical significance. Functional studies have established that miR-129-2 [25], miR-345 [22], and miR-132 [19] are all tumor-suppressive. Verified targets for miR-129-2 (*SOX4* and *CAMTA1*, [25]; *CDK6*, [43]; and *EIF2C3*/*AGO3*, [44]), miR-345 (*BAG3*, [22]), and miR-132 (*HB-EGF* and *TALIN2*, [19]) suggest that silencing of these miRNAs can have important tumorigenic consequences.

Altered expression of miRNAs [45-48] is known to be involved in the multistage colorectal [10] and endometrial tumorigenesis [49], and aberrant DNA methylation which may underlie expression changes can be utilized in preventive and therapeutic interventions. The miRNAome provides a tool for molecular subclassification of cancers for the purposes of diagnosis, prognostic assessment, and treatment. Sporadic MSS-CRCs behave differently compared to their MSI counterparts, and the latter differ from CRCs arising in LS; the differential behavior can at least in part be attributed to epigenetic alterations and their associations with other molecular markers [50]. Differentiation between sporadic MSI, sporadic MSS, and LS-associated CRCs may be possible on the basis of miRNA expression [51-53] and/or methylation patterns ([16] and this study). In our series, MMR-deficient tumors (from LS and sporadic cases) were enriched, and therefore, all clinicopathological correlations we report need to be independently evaluated in large unselected series for confirmation.

## Conclusions

Our investigation provides new insights into colorectal and endometrial tumorigenesis since it (i) links epigenetic inactivation of miR-132 and miR-345 to specific subtypes of colorectal carcinoma, especially with respect to MSI status, (ii) pinpoints miR-129-2 as an important player in the early steps of endometrial tumorigenesis, (iii) shows differential involvement of miRNAs in hereditary *vs*. sporadic cancers, and (iv) provides suggestive evidence to support the role of geographic and/or ethnic origin as a modifier of patterns of miRNA methylation in tumors. The observed miRNA alterations may warrant closer evaluation for biomarker potential for clinical applications.

## Methods

### Patients and samples

This investigation included 205 CRCs and 36 endometrial carcinomas (EC) and paired normal tissues (Table 1). The sporadic CRCs represented consecutive series from Finland [11,54] and Australia [55], with MMR status used as a selection criterion to include MMR-proficient and MMR-deficient tumors in roughly equal proportions. Tumors with unstable *BAT25* or *BAT26* were considered to have MSI, whereas those with normal *BAT25* and *BAT26* were microsatellite-stable (MSS). *BAT25* and *BAT26* are mononucleotide repeat markers from the five-marker Bethesda panel [56] and have been shown to be sensitive and specific indicators of the MSI-high phenotype [57,58]. Immunohistochemical (IHC) analysis of MMR protein expression for Finnish tumors was as described [11] and for Australian tumors conducted by standard methods with MLH1 clone G168-15 (BD Pharmingen 1:100), MSH2 clone 25D12 (Leica 1:250), MSH6 clone BC/44 (Biocare 1:100), and PMS2 clone A16-4 (BD Pharmingen 1:500) as primary antibodies. In addition to the series described in Table 1, an endometrial series [27] including 29 normal endometrial tissues (14 sporadic, 15 Lynch syndrome), 12 simple hyperplasias (10 sporadic, 2 Lynch syndrome), 13 complex hyperplasias without atypia (8 sporadic, 5 Lynch syndrome), and 24 complex hyperplasias with atypia (10 sporadic and 14 Lynch syndrome) was investigated. Following histological evaluation, tumor and hyperplasia samples were procured by appropriate methods to ensure high percentages of tumor and hyperplasia cells [11,27,50,59]. DNA was subsequently extracted from selected regions of FFPE and fresh frozen tumors from Finland using the protocol by Isola *et al*. [60] and from Australian FFPE samples by the Puregene DNA Isolation Kit (Centra, Minneapolis, MN, USA).

The study of Finnish cases was approved by the institutional review board of the Helsinki University Central Hospital (Dnro 466/E6/01) and the National Authority for Medicolegal Affairs (Dnro 1272/04/044/07). For Australian cases, Human Ethics approval was granted by the Ethics Review Committees of Sydney South West Area Health Service (Royal Prince Alfred and Liverpool Hospitals) by protocol numbers X08-0224 and SSA/09/LPOOL/23.

### Drug treatment of cell lines

Five colorectal and two endometrial cancer lines (Additional file 1: Table S1) from the American Type Culture Collection (Rockville, MD, USA) were cultured according to the supplier’s protocol. Demethylation studies were performed as described [61]. Cells were treated with 5 μM 5-aza-2′deoxycytidine (Sigma, A3656; Sigma-Aldrich, St. Louis, MO, USA) for 96 h and with 300 nM trichostatin (Sigma, T1952) for 18 h. All treatments were performed in duplicates. DNA was isolated using standard protocols and total RNA extracted with miRNeasy mini kit (Qiagen, Valencia, CA, USA). The efficiency of the drug treatments was confirmed by expressional (TaqMan®) and methylation (SALSA MS-MLPA ME001-C1 Tumor suppressor-1 kit, MRC-Holland, Amsterdam, the Netherlands) analyses of selected tumor suppressor genes in all cell lines, as well as by IHC analysis of MLH1 in RKO.

### Genome-wide miRNA profiling

Agilent’s human miRNA microarrays (8 × 15 K from Agilent Technologies, G4470B; Agilent Technologies, Inc., Santa Clara, CA, USA) containing 723 human and 76 human viral miRNAs sourced from the Sanger miRBase, v. 10.1 were used. Signal intensities of fluorescence were calculated by Agilent’s Feature Extraction software version 10.7.3.1. Microarrays were investigated in duplicates for each cell line. GeneSpring GX software, version 11.0.2 (Agilent Technologies) was used for miRNA data analysis. Data was normalized by quantile normalization. Statistically significant differentially expressed miRNA were identified by *t*-test unpaired combined with the Benjamini and Hochberg correction for multiple testing and using filters based on *P*-value cutoff 0.05 and fold change cutoff ±2.00. The miRNA expression profiling data have been submitted to GEO (accession number GSE55930).

### CpG island definition and analysis by bisulfite sequencing

The regions of regularly up to 3 kb upstream of the mature miRNAs [62] were screened for CpG islands and the miRNA gene promoter regions defined by the EMBOSS CpGplot (http://www.ebi.ac.uk/Tools/seqstats/emboss_cpgplot) and CpG island searcher (http://ccat.hcs.usc.edu/cpgislands2) programs.

Methylation statuses of the CpG sites in a miRNA-associated CpG island were determined by bisulfite sequencing. In brief, DNAs from cancer cell lines (Additional file 1: Table S1) and normal blood or tissue donors (healthy female and male donor DNA from Promega, Madison, WI, USA, CatG152A and CatG147A; normal colon DNA from Amsbio, Abingdon, UK, LotA805046, and normal uterus DNA from Amsbio, Abingdon, UK, LotB403076) were bisulfite-converted using EZ DNA Methylation-Direct™ Kit (Catalog Number D5021, Zymo Research Corporation, Irvine, CA, USA). Bisulfite-modified DNA was amplified by methylation-unbiased primers (Additional file 4: Table S2) designed with the assistance of MethPrimer-program (http://www.urogene.org/cgi-bin/methprimer/methprimer.cgi) or manually.

Amplification products were sequenced either directly or after cloning. For the latter, amplification products were cloned into a pCR2.1 TOPO vector using the TOPO TA Cloning System (Invitrogen, Carlsbad, CA, USA), and DNAs extracted from the resulting white colonies were sequenced.

### MS-MLPA for methylation studies of miRNAs

CpG dinucleotides that were part of the restriction site for the methylation-sensitive enzyme *HhaI* (GCGC) and with methylation status representative of a larger region as determined by bisulfite sequencing were chosen for the design of probes for custom-made MS-MLPA. In MS-MLPA, a signal peak is generated if the sample DNA is methylated, which protects the DNA probe hybrids against *HhaI* digestion and the ligated probes can be exponentially amplified by PCR. MS-MLPA probes specific for the miRNAs of interest were designed according to the protocol of MRC-Holland (www.MRC-Holland.com) (Additional file 3: Figure S2 and Additional file 5: Table S3).

All selected miRNAs were interrogated by one MS-MLPA probe except for miR-375 with two probes. The synthetic probes were added to the SALSA MLPA P300-A1 Reference-2 kit (MRC-Holland, Amsterdam, the Netherlands) for control probes lacking *Hha1* sites to make a complete MS-MLPA assay. Amplification products were visualized by fragment analysis and methylation dosage ratios (Dm) calculated as described [12].

Custom MS-MLPA assays were optimized and validated by a previously outlined protocol [16,41]. In brief, technical thresholds for a reliable detection of methylation were determined by evaluating MS-MLPA results against bisulfite sequencing. Dm = 0.15 (corresponding to 15% methylated DNA) turned out to be the technical threshold for the present miRNAs based on comparative analyses showing that Dm < 0.15 by MS-MLPA corresponded to unmethylated sequence (T/T) and Dm ≥ 0.15 to partially (C/T) or completely (C/C) methylated sequence by direct bisulfite sequencing. More accurate quantification of methylation to validate the results was obtained by sequencing of cloned bisulfite-converted PCR-amplified fragments as described [16,41]. All seven miRNAs of interest were validated by direct bisulfite sequencing and miR-129-2, miR-345, and miR-132 additionally by cloning. To illustrate the validation procedure, miR-129-2 is shown as an example in Additional file 9: Figure S4.

In this paper, the term *hypermethylation* is used to indicate higher methylation in tumor DNA relative to normal DNA, with miRNA-specific numerical thresholds defined as the average Dm in normal DNA of the same tissue type (colorectal mucosa or normal endometrium) plus one standard deviation (if the calculation resulted in a value below the technical threshold, the technical threshold was used instead) (Additional file 8: Table S5).

### TSG methylator phenotype and CIMP

The TSG methylator phenotype for Finnish tumors was established by the SALSA MS-MLPA ME001-C1 Tumor suppressor-1 kit (MRC-Holland, Amsterdam, The Netherlands) as described [11]. Sporadic MSI CRCs were additionally studied with the SALSA MS-MLPA ME042-B2 CIMP kit (MRC-Holland, Amsterdam, The Netherlands) for verification. The CIMP phenotype for Australian tumors was based on the *CACNA1G*, *IGF2*, *NEURO1G*, *RUNX3*, and *SOCS1* markers [63] and was determined by Methylight analysis of bisulfite-converted DNA (EpiTect Bisulfite Kit, Qiagen, Valencia, CA, USA) as described [17].

### Statistical analyses

Statistical analyses were performed using SPSS Statistics Software (IBM SPSS, Inc., Chicago, IL, USA). Statistical significance for the differences between distributions was evaluated as follows. Depending on whether or not the data were normally distributed (as evaluated by Shapiro-Wilk test), a parametric or non-parametric test, respectively, was chosen. For pairwise analysis of correlated samples (intra-group comparisons), *t*-test (parametric) or Wilcoxon signed rank test (nonparametric) was used. To evaluate the significance of difference between the means of two independent groups (inter-group comparisons), *t*-test (parametric) or Mann-Whitney *U* test (nonparametric) was applied. The percentages of tumors with hypermethylation (Table 2 below) were compared by Fisher Exact Probability Test from VassarStats Web site (www.vassarstats.net/tab2x2.html). For the comparison of multiple (≥3) independent groups, one-way ANOVA and Kruskal-Wallis test were used for parametric and non-parametric analyses, respectively, followed by appropriate *post-hoc* tests. For correlations, the Pearson product-moment correlation coefficient (*r*) for linear correlation was determined for parametric data and the Spearman rank correlation coefficient (rho) for non-parametric data. *P* values <0.05 (two-tailed) were considered significant.

## Additional files

Additional file 1: Table S1. Characteristics of cell lines studied. Additional file 2: Figure S1. Venn diagram showing the number of miRNAs (among all significantly upregulated 109) which were specific to a given group or alternatively, shared between different groups of cancer cell lines. MMR-D EC includes HEC59 and AN3CA, MMR-P CRC includes T84 and SW480, and MMR-D CRC includes HCT15, HCT116, and RKO. The miRNAs associated with CpG islands are underlined, and those selected for this study are in bold. Additional file 3: Figure S2. Schematic figure of the CpG island-containing region associated with each miRNA. The locations of the MS-MLPA probes (gray boxes) and the primers for bisulfite sequencing (BS, black arrowheads) are given. The location of the mature miRNA is shown by a thin arrow which indicates transcriptional direction. Additional file 4: Table S2. Sequences for bisulphite sequencing primers. Additional file 5: Table S3. Sequences for MS-MLPA probes. Additional file 6: Table S4. Average methylation dosage ratios and standard deviations for paired tumor and normal tissues shown in Figure 3. Additional file 7: Figure S3. Percentages of tumors with hypermethylation relative to the respective normal tissues at the seven individual miRNA loci. The exact percentage is given above each bar. Please see Additional file 8: Table S5 for cut-off values for hypermethylation and Table 2 for statistical analysis of group-specific comparisons. Additional file 8: Table S5. Normal tissue-based threshold values for the detection of hypermethylation at miRNA loci in tumor tissues. Additional file 9: Figure S4. The design and validation of MS-MLPA assays showing miR-129-2 as an example. Bisulphite-converted DNA from cancer cell lines and normal tissues was first sequenced to select a representative region for MS-MLPA probe design (see Methods). Results from direct bisulphite sequencing (without cloning) are depicted on the left, with methylation status of each CpG site coded as T/T (unmethylated), C/T (partially methylated), or C/C (methylated). Quantification of DNA methylation (proportion of methylated DNA) by two parallel methods, MS-MLPA and sequencing of cloned bisulphite-converted PCR-amplified fragments, is shown on the right. Dm values by MS-MLPA were concordant with results from the cloning analysis.

## Abbreviations

5-AZA-CdR: 5-aza-2′ deoxycytidine
CAH: complex hyperplasia with atypia
CH: complex hyperplasia without atypia
CIMP: CpG island methylator phenotype
CIN: chromosomal instability
LS: Lynch syndrome
MMR: mismatch repair
MSI: microsatellite instability
MS-MLPA: methylation-specific multiplex ligation-dependent probe amplification
MSS: microsatellite stable
TSA: trichostatin A
TSG: tumor suppressor gene
