# Supplementary material for: Identification of subgroup-specific miRNA patterns by epigenetic profiling of sporadic and Lynch syndrome-associated colorectal and endometrial carcinoma
Source: Clin Epigenetics. 2015 Mar 10;7(1):20. doi: 10.1186/s13148-015-0059-3 (PMC4357086; doi:10.1186/s13148-015-0059-3)
Supplement: Additional file 1: Table S1. — Characteristics of cell lines studied. [file 13148_2015_59_MOESM1_ESM.pdf]

Supplementary Table1. Characteristics of cell lines studied

Cell line	Cancer type	MMR status	Ref	CIMP	Ref
HCT15	colon	deficient (homozygous *MSH6* mutation)	[1, 2]	high	[3]
HCT116	colon	deficient (hemizygous *MLH1* mutation)	[4]	high	[3]
RKO	colon	deficient (biallelic *MLH1* methylation)	[5]	high	[3]
T84	colon	proficient	[6, 7]	low	This study
SW480	colon	proficient	[8, 7]	low	This study
HEC59	endometrial	deficient (*MSH2* biallelic mutations)	[1, 9]	high	[3]
AN3CA	endometrial	deficient (*MLH1* biallelic methylation)	[5, 10]	high	[3]

[1] Boyer JC, Umar A, Risinger JI, Lipford JR, Kane M, Yin S, Barrett JC, Kolodner RD, Kunkel TA: **Microsatellite instability, mismatch repair deficiency, and genetic defects in human cancer cell lines.** *Cancer Res* 1995, **55**:6063-6070.
[2] Papadopoulos N, Nicolaides NC, Liu B, Parsons R, Lengauer C, Palombo F, D'Arrigo A, Markowitz S, Willson JKV, Kinzler KW, Jiricny J, Vogelstein B: **Mutations of GTBP in genetically unstable cells.** *Science* 1995, **268**:1915-1917.
[3] Joensuu EI, Abdel-Rahman WM, Ollikainen M, Ruosaari S, Knuutila S, Peltomäki P: **Epigenetic signatures of familial cancer are characteristics of tumor type and family category.** *Cancer Res* 2008, **68**:4597-4605.
[4] Papadopoulos N, Nicolaides NC, Wei YF, Ruben SM, Carter KC, Rosen CA, Haseltine WA, Fleischmann RD, Fraser CM, Adams MD, Venter JC, Hamilton SR, Petersen GM, Watson P, Lynch HT, Peltomäki P, Mecklin J-P, de la Chapelle A, Kinzler KW, Vogelstein B: **Mutation of a mutL homolog in hereditary colon cancer.** *Science* 1994, **263**:1625-1629.
[5] Veigl ML, Kasturi L, Olechnowicz J, Ma AH, Lutterbaugh JD, Periyasamy S, Li GM, Drummond J, Modrich PL, Sedwick WD, Markowitz SD: **Biallelic inactivation of hMLH1 by epigenetic gene silencing, a novel mechanism causing human MSI cancers.** *Proc Natl Acad Sci USA* 1997, **95**:8698-8702.
[6] Knutsen T, Padilla-Nash HM, Wangsa D, Barenboim-Stapleton L, Camps J, McNeil N, Difilippantonio MJ, Ried T: **Defective molecular cytogenetic characterization of 15 colorectal cancer cell lines.** *Genes Chromosomes Cancer* 2010, **49**:204-223.
[7] Suter CM, Norrie M, Ku SL, Cheong KF, Tomlinson I, Ward RL: **CpG island methylation is a common finding in colorectal cancer cell lines.** *Br J Cancer* 2003, **88**:413-419.
[8] Kleivi K, Teixeira MR, Eknaes M, Diep CB, Jakobsen KS, Hamelin R, Lothe RA: **Genome signatures of colon carcinoma cell lines.** *Cancer Genet Cytogenet* 2004, **155**:119-131.
[9] Umar A, Koi M, Risinger JI, Glaab WE, Tindall KR, Kolodner RD, Boland CR, Barrett JC, Kunkel TA: **Correction of hypermutability, N-methyl-N'-nitro-N-nitrosoguanidine resistance, and defective DNA mismatch repair by introducing chromosome 2 into human tumor cells with mutations in MSH2 and MSH6.** *Cancer Res* 1997, **57**:3949-3955.
[10] Kane MF, Loda M, Gaida GM, Lipman J, Mishra R, Goldman H, Jessup JM, Kolodner R: **Methylation of the hMLH1 promoter correlates with lack of expression of hMLH1 in sporadic colon tumors and mismatch repair-defective human tumor cell lines.** *Cancer Res* 1997, **57**:808-811.
