# Supplementary material for: Identification of subgroup-specific miRNA patterns by epigenetic profiling of sporadic and Lynch syndrome-associated colorectal and endometrial carcinoma
Source: Clin Epigenetics. 2015 Mar 10;7(1):20. doi: 10.1186/s13148-015-0059-3 (PMC4357086; doi:10.1186/s13148-015-0059-3)

Supplementary Figure 1.

Venn diagram showing the number of miRNAs (among all significantly upregulated 109) which were specific to a given group or alternatively, shared between different groups of cancer cell lines. MMR-D EC includes HEC59 and AN3CA, MMR-P CRC includes T84 and SW480, and MMR-D CRC includes HCT15, HCT116, and RKO.

The miRNAs associated with CpG islands are underlined, and those selected for this study are in bold.

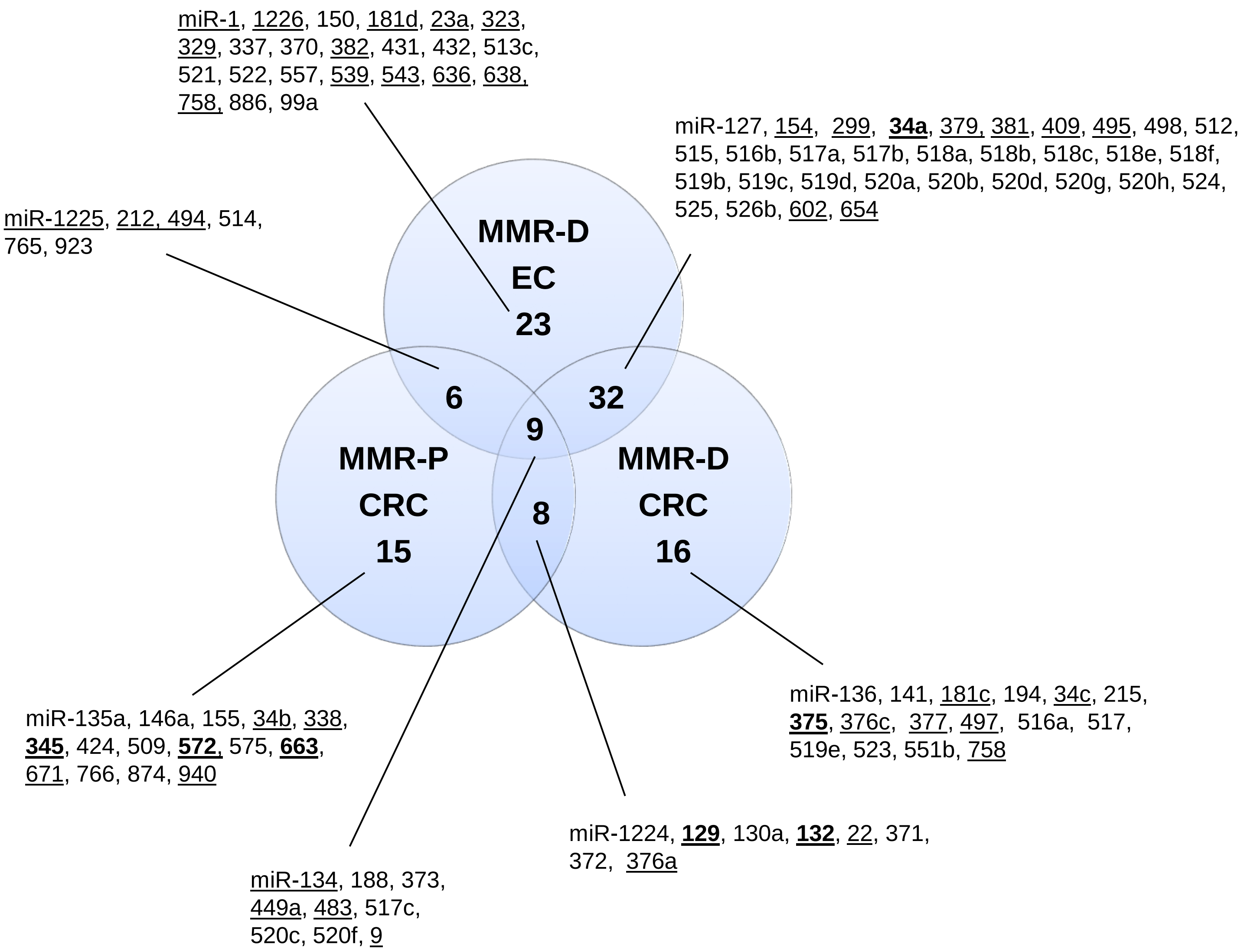

Supplement: Additional file 2: Figure S1. — Venn diagram showing the number of miRNAs (among all significantly upregulated 109) which were specific to a given group or alternatively, shared between different groups of cancer cell lines. MMR-D EC includes HEC59 and AN3CA, MMR-P CRC includes T84 and SW480, and MMR-D CRC includes HCT15, HCT116, and RKO. The miRNAs associated with CpG islands are underlined, and those selected for this study are in bold. [file 13148_2015_59_MOESM2_ESM.pdf]
