# Supplementary material for: Identification of subgroup-specific miRNA patterns by epigenetic profiling of sporadic and Lynch syndrome-associated colorectal and endometrial carcinoma
Source: Clin Epigenetics. 2015 Mar 10;7(1):20. doi: 10.1186/s13148-015-0059-3 (PMC4357086; doi:10.1186/s13148-015-0059-3)
Supplement: Additional file 3: Figure S2. — Schematic figure of the CpG island-containing region associated with each miRNA. The locations of the MS-MLPA probes (gray boxes) and the primers for bisulfite sequencing (BS, black arrowheads) are given. The location of the mature miRNA is shown by a thin arrow which indicates transcriptional direction. [file 13148_2015_59_MOESM3_ESM.pdf]

Supplementary Figure 2. Schematic figure of the CpG island-containing region associated with each miRNA. The locations of the MS-MLPA probes (gray boxes) and the primers for bisulfite sequencing (BS, black arrowheads) are given. The location of the mature miRNA is shown by a thin arrow which indicates transcriptional direction.

mir- 129-2 (CpG island 1 start=338, end=1811. CpG island 2 start=2332, end=3090)

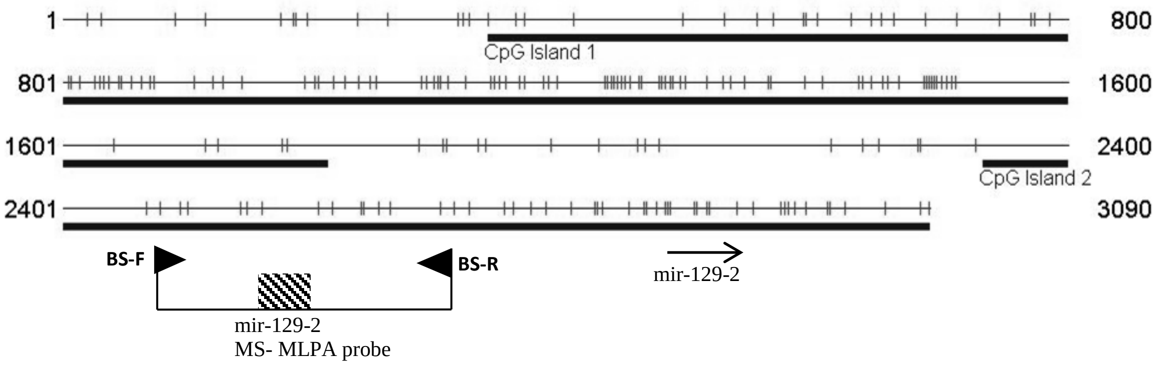

Bandres E, Agirre X, Bitarne N, Ramirez R, Zarate R, Roman-Gomez J, Prosper F, Garcia-Foncillas J: **Epigenetic regulation of microRNA expression in colorectal cancer.** *Int J Cancer* 2009, **125**:2737-2743.

mir-572 (CpG island 1 start=2540, end 3155)

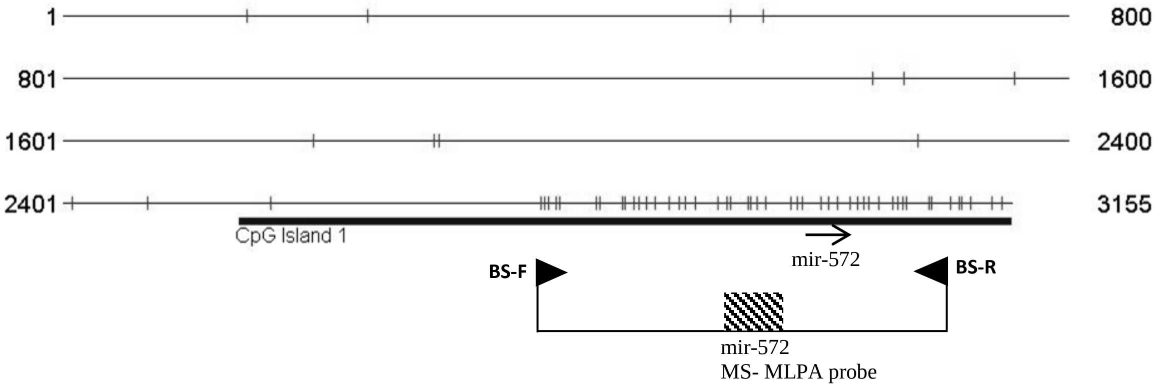

Suzuki H, Takatsuka S, Akashi H, Yamamoto E, Nojima M, Maruyama R, Kai M, Yamano HO, Sasaki Y, Tokino T, Shinomura Y, Imai K, Toyota M: **Genome-wide Profiling of Chromatin Signatures Reveals Epigenetic Regulation of MicroRNA Genes in Colorectal Cancer.** *Cancer Res* 2011, **71**:5646-5658.

mir-663 (CpG island 1 start=1191, end 3093)

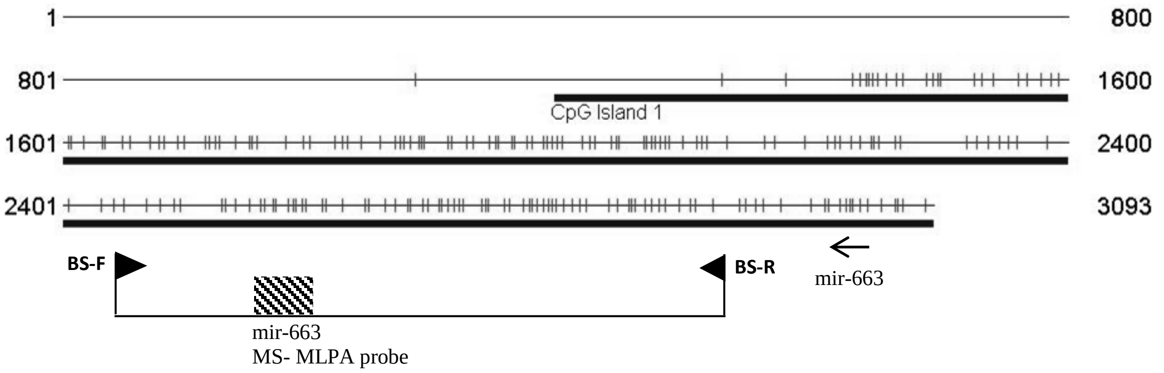

Lehmann U, Hasemeier B, Christgen M, Müller M, Römermann D, Länger F, Kreipe H: **Epigenetic inactivation of microRNA gene *hsa-mir-9-1* in human breast cancer.** *J Pathol* 2008, **214**:17-24.

mir-375-I and mir 375-II (CpG island 1 start=1015, end 3420)

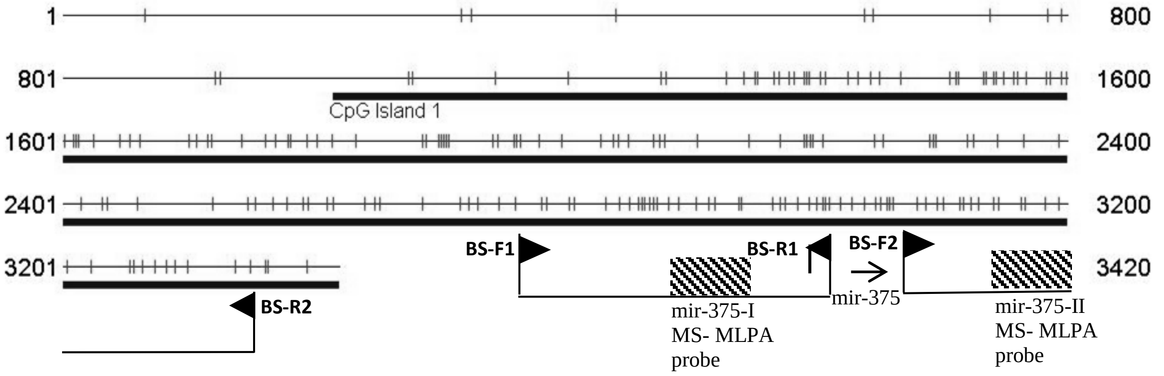

de Souza Rocha Simonini P, Breiling A, Gupta N, Malekpour M, Omranipour R, Makekpour F, Youns M, Omranipour R, Malekpour F, Volinia S, Croce CM, Najmabadi H, Diederichs S, Sahin Ö, Mayer D, Lyko F, Hoheisel JD, Riazalhosseini Y: **Epigenetically Deregulated microRNA-375 Is Involved in a Positive feedback Loop with Estrogen Receptor α in Breast Cancer Cells.** *Cancer Res* 2010, **70**:9175-9184.

mir-345 (CpG island 1 start=191, end=2688)

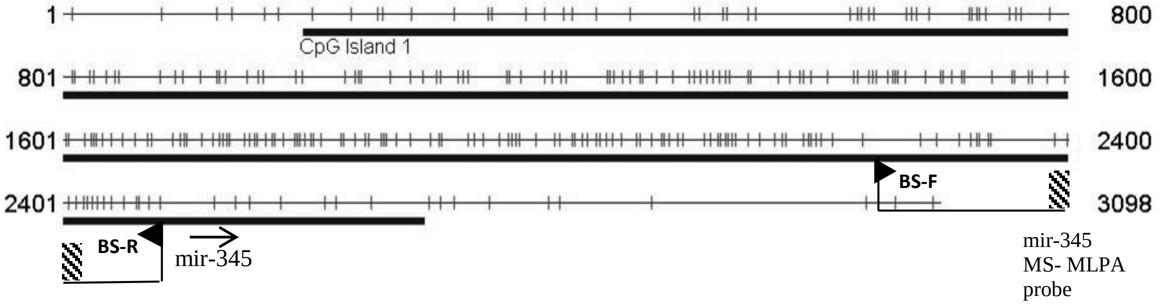

Tang J-T, Wang J-L, Du W, Hong J, Zhao SL, Wang YC, Xiong H, Chen HM, Fang JY: **MicroRNA 345, a methylation-sensitive microRNA is involved in cell proliferation and invasion in human colorectal cancer.** *Carcinogenesis* 2011, **32**:1207-1215.

mir-132 (CpG island 1 start=1, end=642. CpG island 2 start=2054, end=4001) 4kb upstream

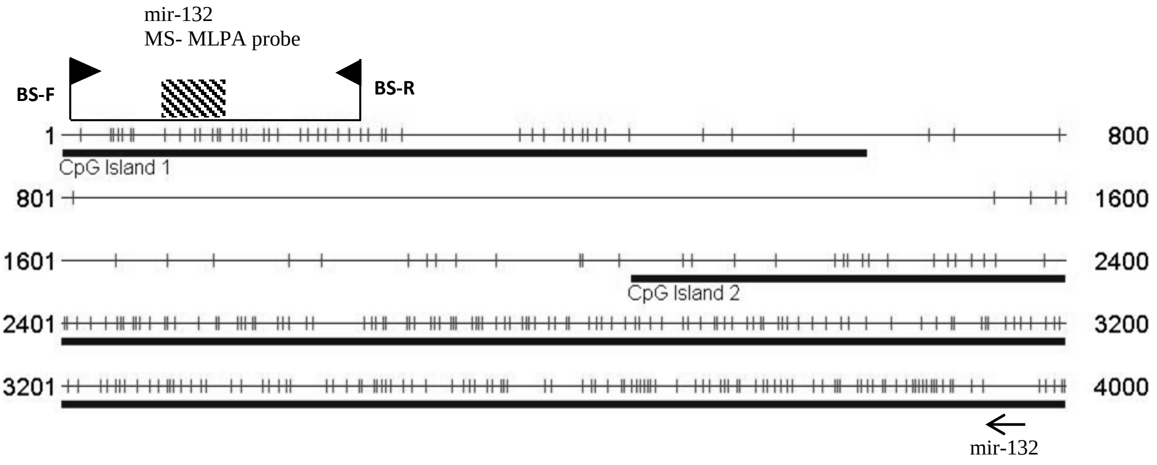

Formosa A, Lena AM, Markert EK, Cortelli S, Miano R, Mauriello A, Croce N, Vandesompele J, Mestdagh P, Finazzi-Agro E, Levine AJ, Melino G, Bernardini S, Candi E: **DNA methylation silences miR-132 in prostate cancer.** *Oncogene* 2013, **32**:127-134.

mir 34a (CpG island start=1, end=1735, ~32kb upstream of mature miRNA)

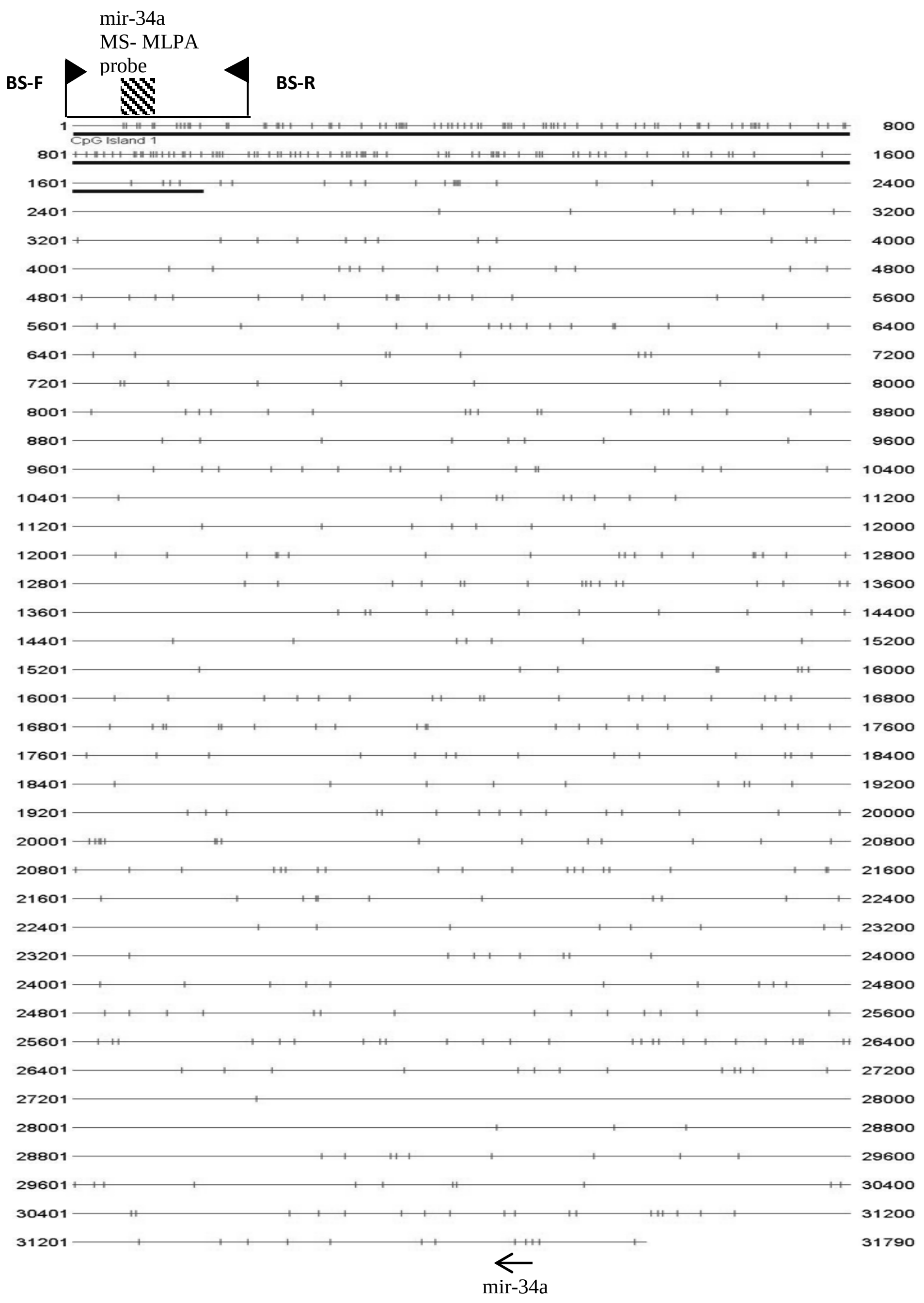

Lodygin D, Tarasov V, Epanchintsev A, Berking C, Knyazeva T, Körner H, Knyazev P, Diebold J, Hermeking H: **Inactivation of miR-34a by aberrant CpG methylation in multiple types of cancer.** *Cell Cycle* 2008, **7**:2591-2600.
