# Supplementary material for: Identification of subgroup-specific miRNA patterns by epigenetic profiling of sporadic and Lynch syndrome-associated colorectal and endometrial carcinoma
Source: Clin Epigenetics. 2015 Mar 10;7(1):20. doi: 10.1186/s13148-015-0059-3 (PMC4357086; doi:10.1186/s13148-015-0059-3)
Supplement: Additional file 4: Table S2. — Sequences for bisulphite sequencing primers. [file 13148_2015_59_MOESM4_ESM.pdf]

Supplementary Table 2. Sequences for bisulphite sequencing primers

miRNA	Reference	Sequence (5`- 3`)	Product	Ta[a]
miR-572_F1	This study	GAATTATATTTGGTAAAAAAAATTAAAATAGAG	366	52-54
miR-572_R1	This study	CCCCRCTAATCTAAATCCCRCRAAC		
miR-129-2_F2	This study	GAAAAAAAAGGGGGAAATGTGAG	329	54-56
miR-129-2_R2	This study	AACCCAACCTCCACCCCTTTCCT		
miR-663_F	[1]	GTTTGTAGAGGAATTTTTTTTAGTT	440	56-58
miR-663_R	[1]	ACCACAACCACAAACTCAAC		
miR-375_F1	This study	GGGATTGAATAGGTAGTATAAGAGTATA	319	52-54
miR-375_R1	This study	AAACRAAAACRAAAAACCCRAAACCTAA		
miR-375_F2	This study	GAGGTAGGGGYGGTTTTTTAGTATTAG	283	52-54
miR-375_R2	This study	TTCCACCTCCAAAAAAATTTCTATTCT		
miR-345_F	[2]	GGTTTTTGGATTGGGTTGTAGAGTG	239	56-58
miR-345_R	[2]	AACCAAAACAATCCCTTACCACTAC		
miR-132_F	This study	TGGGATTTTATTGTTTTTAAAGTGAG	296	56-58
miR-132_R	This study	CTATCCTCTAACCCCCATAACC		
miR-34a_F	[3]	GGGTAYGGTGGTTTTGGGGGTAGTTTG	250	64
miR-34a_R1	This study	CCTTGGGGGCTGCCCCATGCTCCCCCCTGTAG		

[a] Annealing temperature

[1] Lehmann U, Hasemeier B, Christgen M, Müller M, Römermann D, Länger F, Kreipe H: **Epigenetic inactivation of microRNA gene has-mir-9-1 in human breast cancer.** *J Pathol* 2008, **214**:17-24.

[2] Tang JT, Wang JL, Du W, Hong J, Zhao SL, Wang YC, Xiong H, Chen HM, Fang JY: **MicroRNA 345, a methylation-sensitive microRNA is involved in cell proliferation and invasion in human colorectal cancer.** *Carcinogenesis* 2011, **32**:1207-1215.

[3] Lodygin D, Tarasov V, Epanchintsev A, Berking C, Knyazeva T, Körner H, Knyazev P, Diebold J, Hermeking H: **Inactivation of miR-34a by aberrant CpG methylation in multiple types of cancer.** *Cell Cycle* 2008, **7**: 2591-2600.
