# Supplementary material for: Identification of subgroup-specific miRNA patterns by epigenetic profiling of sporadic and Lynch syndrome-associated colorectal and endometrial carcinoma
Source: Clin Epigenetics. 2015 Mar 10;7(1):20. doi: 10.1186/s13148-015-0059-3 (PMC4357086; doi:10.1186/s13148-015-0059-3)
Supplement: Additional file 5: Table S3. — Sequences for MS-MLPA probes. [file 13148_2015_59_MOESM5_ESM.pdf]

Supplementary Table 3. Sequences for MS-MLPA probes

| miRNA | | MS-MLPA probes (5´-3´) | Product size (bp) | Distance of *Hha1* site to mature miRNA (bp) | GC% |
|---|---|---|---|---|---|
| 572 | LPO | GGGTTCCCTAAGGGTTGGA GCCCGGGGCGG**GCGC**ATTAGGAGGTGT | 97 | 94 | 67 |
| | RPO | CGAGGCCGTGGCCCGGAAGTGGTCGGGG TCTAGATTGGATCTTGCTGGCAC | | | 65 |
| 129-2 | LPO | GGGTTCCCTAAGGGTTGGA CTTTACGCA**GCGC**CTGTAGTCACGGCTGGAA | 102 | 491 | 57 |
| | RPO | CCTTGGCTGGGGGACTCTGAGGGATAAGA TCTAGATTGGATCTTGCTGGCAC | | | 60 |
| 663 | LPO | GGGTTCCCTAAGGGTTGGA CATCTTGAGTC CCTACTGTGGCCG**GCGC**CTCCCCTTTC | 114 | 429 | 70 |
| | RPO | CGAGTAAGGGGAGGATCCCGCCGGCAG TTCATGG TCTAGATTGGATCTTGCTGGCAC | | | 70 |
| 375-I | LPO | GGGTTCCCTAAGGGTTGGA CATCTTGAGTC CATCTTGAG GCGGAGGCTAGCGGG**GCGC**TGTGCAGCACTGA | 133 | 143 | 72 |
| | RPO | GCTCGCGGAAGACCAGGACCAGGAGATCACCG TTCATGG TCTAGATTGGATCTTGCTGGCAC | | | 66 |
| 375-II | LPO | GGGTTCCCTAAGGGTTGGA CATCTTGAGTC CATCTTGAGTC CAT GCCACCGCCATCTCAACCGTACGGGTGGG | 139 | 135 | 69 |
| | RPO | AGAGGCTGT**GCGC**CGCTCCAGGGGAGATC TTCATGG TTCATGG TCTAGATTGGATCTTGCTGGCAC | | | 69 |
| 345 | LPO | GGGTTCCCTAAGGGTTGGA CATCTTGAGTC CATCTTGAGTC CATCTTGAG AGGGGCC**GCGC**TCAGGCGCTGAAGTCGGA | 152 | 573 | 50 |
| | RPO | GGGTGCTTGGCTCTGGGTCACCAGGGTAT TTCATGG TTCATGG TTCATGG TCTAGATTGGATCTTGCTGGCAC | | | 52 |
| 132 | LPO | GGGTTCCCTAAGGGTTGGA CATCTTGAGTCCATCTTGAGTCCATCTTGA GCTACCAGCGGCCCCCGAGCGACACCAA | 158 | 3817 | 71 |
| | RPO | GGTCTCGG**GCGC**CCCCCGGAGGCTCCCTTT TTCATGGTTCATGGTTCATGGTTCATGG TCTAGATTGGATCTTGCTGGCAC | | | 77 |
| 34a | LPO | GGGTTCCCTAAGGGTTGGA CATCTTGAGTC CATCTTGAGTC CATCTTGAGTC CATCTTGAGTC CTGGGTGGGGAGCCCCTTCTCCATGG | 165 | 31656 | 68 |
| | RPO | CGGCGGGAGGTCTTCG**GCGC**TGCCC TTCATGG TTCATGG TTCATGG TTCATGG TCTAGATTGGATCTTGCTGGCAC | | | 80 |

LPO, left probe oligonucleotide, RPO, right probe oligonucleotide

*HhaI* site in **bold**
