# Supplementary material for: Identification of subgroup-specific miRNA patterns by epigenetic profiling of sporadic and Lynch syndrome-associated colorectal and endometrial carcinoma
Source: Clin Epigenetics. 2015 Mar 10;7(1):20. doi: 10.1186/s13148-015-0059-3 (PMC4357086; doi:10.1186/s13148-015-0059-3)
Supplement: Additional file 6: Table S4. — Average methylation dosage ratios and standard deviations for paired tumor and normal tissues shown in Figure 3. [file 13148_2015_59_MOESM6_ESM.pdf]

Supplementary Table 4.

Average methylation dosage ratios and standard deviations for paired tumor and normal tissues shown in Fig 3.

Tissue category	No.	572	129-2	663	375	345	132	34a
Sporadic Finnish CRC-MSI	40	0.56	0.36	0.42	0.04	0.26	0.20	0.60
		(0.09)‡	(0.12)	(0.11)	(0.03)	(0.16)	(0.19)	(0.16)
Normal mucosa	40	0.51	0.16	0.30	0.04	0.11	0.02	0.44
		(0.08)	(0.05)	(0.08)	(0.03)	(0.04)	(0.02)	(0.14)
p-value[a]		**<0.05**	**<0.001**	**<0.001**	**ns**	**<0.001**	**<0.001**	**<0.001**
Sporadic Finnish CRC-MSS	47	0.51	0.30	0.37	0.04	0.13	0.03	0.52
		(0.15)	(0.14)	(0.14)	(0.03)	(0.07)	(0.08)	(0.16)
Normal mucosa	47	0.45	0.15	0.25	0.05	0.10	0.03	0.41
		(0.14)	(0.05)	(0.10)	(0.04)	(0.05)	(0.03)	(0.13)
p-value		**<0.01**	**<0.001**	**<0.001**	**ns**	**<0.001**	**ns**	**<0.001**
Finnish Lynch-CRC	28	0.60	0.30	0.41	0.12	0.21	0.07	0.56
		(0.13)	(0.11)	(0.12)	(0.05)	(0.15)	(0.04)	(0.15)
Normal mucosa		0.52	0.20	0.23	0.13	0.16	0.07	0.39
		(0.12)	(0.06)	(0.08)	(0.09)	(0.07)	(0.05)	(0.16)
p-value		**<0.05**	**<0.001**	**<0.001**	**ns**	**ns**	**ns**	**<0.001**
Sporadic Australian CRC-MSI	38	0.63	0.44	0.53	0.13	0.24	0.25	0.79
		(0.15)	(0.17)	(0.14)	(0.08)	(0.22)	(0.22)	(0.31)
Normal mucosa		0.55	0.22	0.33	0.10	0.13	0.07	0.51
		(0.20)	(0.06)	(0.13)	(0.06)	(0.06)	(0.04)	(0.30)
p-value		**<0.001**	**<0.01**	**<0.001**	**ns**	**<0.01**	**<0.001**	**<0.001**
Sporadic Australian CRC-MSS	52	0.64	0.49	0.54	0.13	0.15	0.12	0.75
		(0.11)	(0.19)	(0.12)	(0.04)	(0.13)	(0.12)	(0.22)
Normal mucosa		0.58	0.27	0.31	0.12	0.16	0.11	0.67
		(0.10)	(0.06)	(0.09)	(0.05)	(0.10)	(0.14)	(0.52)
p-value		**<0.05**	**<0.001**	**<0.001**	**ns**	**ns**	**ns**	**<0.01**
Finnish Lynch-EC	36	0.43	0.22	0.31	0.10	0.08	0.12	0.34
		(0.16)	(0.10)	(0.12)	(0.06)	(0.05)	(0.10)	(0.13)
Normal endometrium		0.32	0.15	0.15	0.09	0.09	0.06	0.22
		(0.12)	(0.06)	(0.11)	(0.06)	(0.05)	(0.05)	(0.07)
p-value		**<0.01**	**<0.001**	**<0.001**	**ns**	**ns**	**<0.01**	**<0.001**

‡Number in parentheses, standard deviation

[a]Statistical significance for tumor vs. normal comparisons was determined with T-test (pairwise analysis) or Wilcoxon test and significant values are in bold.

ns, non-significant
