# Supplementary material for: Identification of subgroup-specific miRNA patterns by epigenetic profiling of sporadic and Lynch syndrome-associated colorectal and endometrial carcinoma
Source: Clin Epigenetics. 2015 Mar 10;7(1):20. doi: 10.1186/s13148-015-0059-3 (PMC4357086; doi:10.1186/s13148-015-0059-3)
Supplement: Additional file 7: Figure S3. — Percentages of tumors with hypermethylation relative to the respective normal tissues at the seven individual miRNA loci. The exact percentage is given above each bar. Please see Additional file 8: Table S5 for cut-off values for hypermethylation and Table 2 for statistical analysis of group-specific comparisons. [file 13148_2015_59_MOESM7_ESM.pdf]

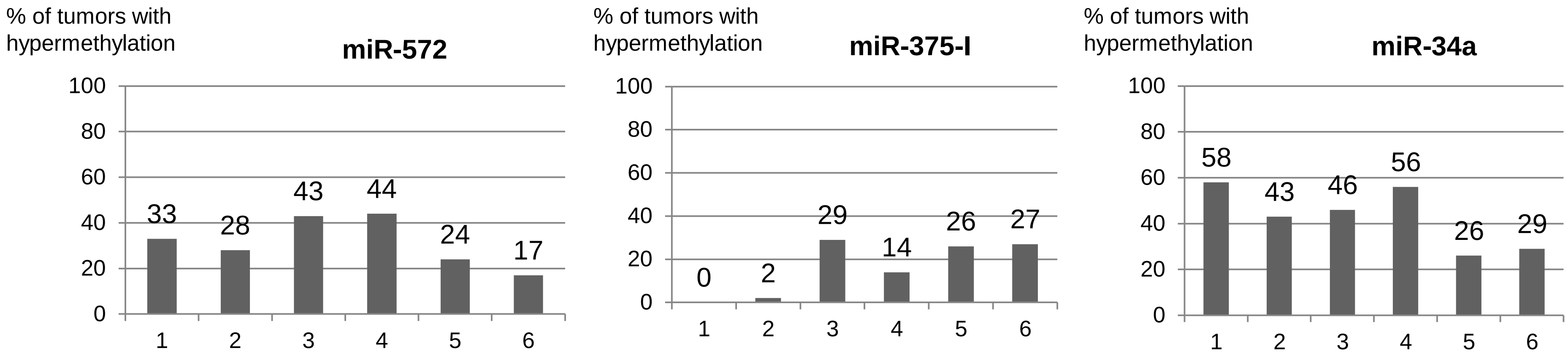

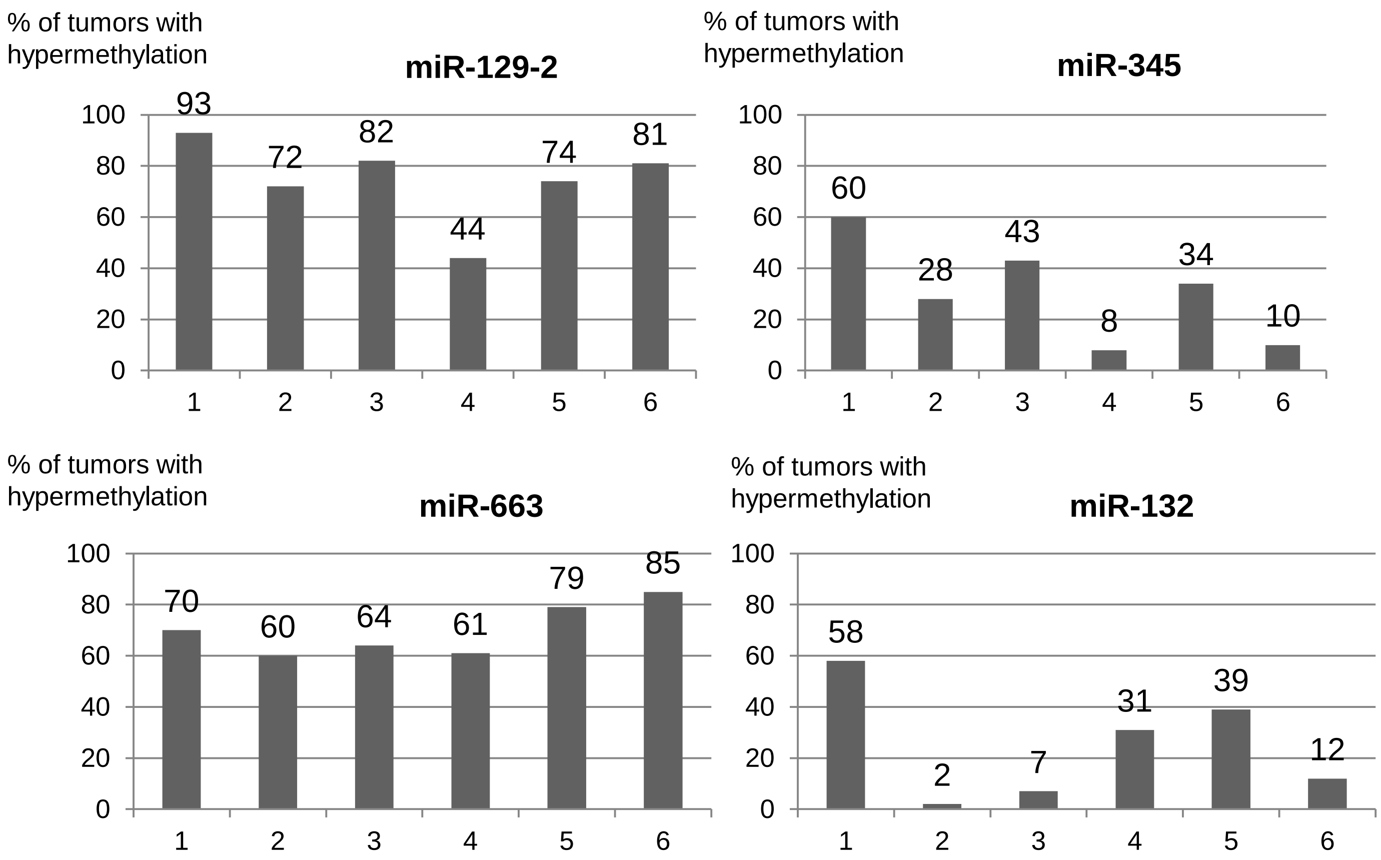

1. Sporadic Finnish MSI-CRC
2. Sporadic Finnish MSS-CRC
3. Finnish Lynch-CRC
4. Finnish Lynch-EC
5. Sporadic Australian MSI-CRC
6. Sporadic Australian MSS-CRC

Supplementary Figure 3. Percentages of tumors with hypermethylation relative to the respective normal tissues at the seven individual miRNA loci. The exact percentage is given above each bar. Please see Supplementary Table 5 for cut-off values for hypermethylation and Table 2 for statistical analysis of group-specific comparisons.
