# Supplementary material for: Identification of subgroup-specific miRNA patterns by epigenetic profiling of sporadic and Lynch syndrome-associated colorectal and endometrial carcinoma
Source: Clin Epigenetics. 2015 Mar 10;7(1):20. doi: 10.1186/s13148-015-0059-3 (PMC4357086; doi:10.1186/s13148-015-0059-3)
Supplement: Additional file 8: Table S5. — Normal tissue-based threshold values for the detection of hypermethylation at miRNA loci in tumor tissues. [file 13148_2015_59_MOESM8_ESM.pdf]

Supplementary Table 5.
Normal tissue-based threshold values for the detection of hypermethylation at miRNA loci in tumor tissues.

Hypermethylation						
miRNA	Normal tissue		Average methylation dosage ratio ($\bar{x}_{Dm}$)	Standard deviation	$\bar{x}_{Dm}$+ 1 standard deviation	Threshold value used‡
572	colorectal	Finnish CRCs	0.49	0.12	0.61	0.61
	colorectal	Australian CRCs	0.57	0.15	0.72	0.72
	endometrial	Lynch EC	0.32	0.12	0.44	0.44
	endometrial	sporadic hyperplasias	0.45	0.09	0.54	0.54
	endometrial	Lynch hyperplasias&EC	0.56	0.18	0.74	0.74
129-2	colorectal	Finnish CRCs	0.16	0.06	0.22	0.22
	colorectal	Australian CRCs	0.25	0.06	0.31	0.31
	endometrial	Lynch EC	0.15	0.06	0.21	0.21
	endometrial	sporadic hyperplasias	0.15	0.04	0.19	0.19
	endometrial	Lynch hyperplasias&EC	0.20	0.09	0.29	0.29
663	colorectal	Finnish CRCs	0.26	0.09	0.35	0.35
	colorectal	Australian CRCs	0.32	0.10	0.42	0.42
	endometrial	Lynch EC	0.15	0.11	0.26	0.26
	endometrial	sporadic hyperplasias	0.20	0.05	0.25	0.25
	endometrial	Lynch hyperplasias&EC	0.23	0.11	0.34	0.34
375-I	colorectal	Finnish CRCs	0.07	0.06	0.13	0.15[a]
	colorectal	Australian CRCs	0.11	0.05	0.16	0.16
	endometrial	Lynch EC	0.09	0.06	0.15	0.15
	endometrial	sporadic hyperplasias	0.04	0.03	0.07	0.15[a]
	endometrial	Lynch hyperplasias&EC	0.12	0.06	0.18	0.18
345	colorectal	Finnish CRCs	0.12	0.06	0.18	0.18
	colorectal	Australian CRCs	0.15	0.08	0.23	0.23
	endometrial	Lynch EC	0.09	0.05	0.14	0.15[a]
	endometrial	sporadic hyperplasias	0.04	0.03	0.07	0.15[a]
	endometrial	Lynch hyperplasias&EC	0.09	0.06	0.15	0.15
132	colorectal	Finnish CRCs	0.03	0.04	0.07	0.15[a]
	colorectal	Australian CRCs	0.09	0.11	0.20	0.20
	endometrial	Lynch EC	0.06	0.05	0.11	0.15[a]
	endometrial	sporadic hyperplasias	0.04	0.03	0.07	0.15[a]
	endometrial	Lynch hyperplasias&EC	0.10	0.11	0.21	0.21
34a	colorectal	Finnish CRCs	0.42	0.14	0.56	0.56
	colorectal	Australian CRCs	0.56	0.28	0.84	0.84
	endometrial	Lynch EC	0.22	0.07	0.29	0.29
	endometrial	sporadic hyperplasias	0.34	0.09	0.43	0.43
	endometrial	Lynch hyperplasias&EC	0.45	0.19	0.64	0.64

‡ $\bar{x}_{Dm}$ + 1 SD or technical threshold (Dm = 0.15)[a], whichever was higher
