# Supplementary material for: Identification of subgroup-specific miRNA patterns by epigenetic profiling of sporadic and Lynch syndrome-associated colorectal and endometrial carcinoma
Source: Clin Epigenetics. 2015 Mar 10;7(1):20. doi: 10.1186/s13148-015-0059-3 (PMC4357086; doi:10.1186/s13148-015-0059-3)
Supplement: Additional file 9: Figure S4. — The design and validation of MS-MLPA assays showing miR-129-2 as an example. Bisulphite-converted DNA from cancer cell lines and normal tissues was first sequenced to select a representative region for MS-MLPA probe design (see Methods). Results from direct bisulphite sequencing (without cloning) are depicted on the left, with methylation status of each CpG site coded as T/T (unmethylated), C/T (partially methylated), or C/C (methylated). Quantification of DNA methylation (proportion of methylated DNA) by two parallel methods, MS-MLPA and sequencing of cloned bisulphite-converted PCR-amplified fragments, is shown on the right. Dm values by MS-MLPA were concordant with results from the cloning analysis. [file 13148_2015_59_MOESM9_ESM.pdf]

Supplementary Figure 4. miR-129-2

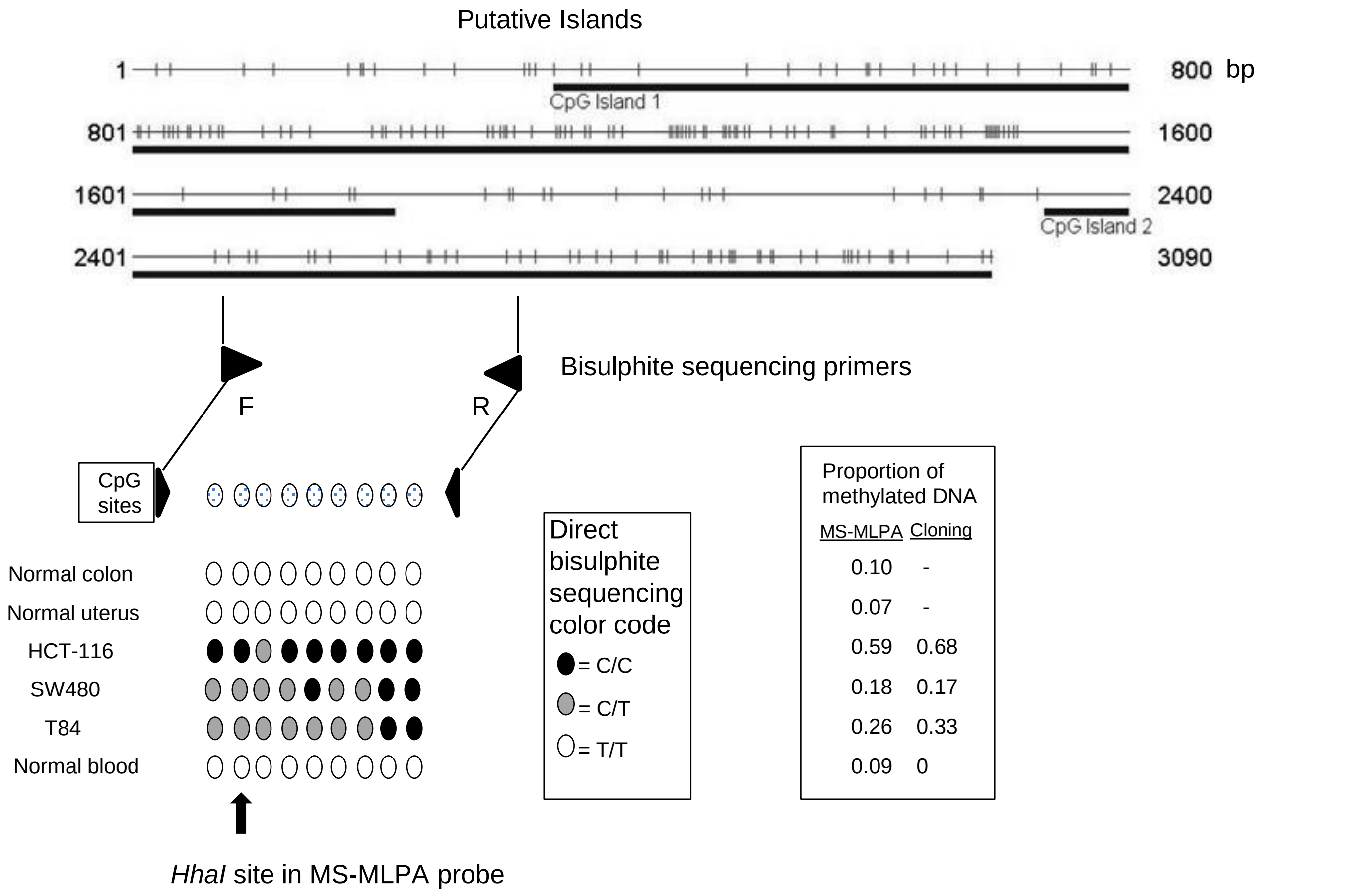

Supplementary Figure 4. The design and validation of MS-MLPA assays showing miR-129-2 as an example. Bisulphite-converted DNA from cancer cell lines and normal tissues was first sequenced to select a representative region for MS-MLPA probe design (see Methods). Results from direct bisulphite sequencing (without cloning) are depicted on the left, with methylation status of each CpG site coded as T/T (unmethylated), C/T (partially methylated), or C/C (methylated). Quantification of DNA methylation (proportion of methylated DNA) By two parallel methods, MS-MLPA and sequencing of cloned bisulphite-converted PCR-amplified fragments, is shown on the right. Dm values by MS-MLPA were concordant with results from the cloning analysis.
